# The potential climate benefits of seaweed farming in temperate waters

**DOI:** 10.1038/s41598-024-65408-3

**Published:** 2024-07-01

**Authors:** Cameron D. Bullen, John Driscoll, Jenn Burt, Tiffany Stephens, Margot Hessing-Lewis, Edward J. Gregr

**Affiliations:** 1 SciTech Environmental Consulting, 2136 Napier Street, Vancouver, BC Canada V5L 2N9; 2 Institute for Resources, Environment and Sustainability, University of British Columbia, Vancouver, BC, Canada; 3 Nature United, North Vancouver, BC Canada; 4 College of Fisheries and Ocean Sciences, University of Alaska Fairbanks, Juneau, AK USA; 5 Hakai Institute, Campbell River, BC Canada; 6 Institute for the Oceans and Fisheries, University of British Columbia, Vancouver, BC Canada

**Keywords:** Climate-change ecology, Ecosystem services, Systems analysis, Climate-change mitigation

## Abstract

Seaweed farming is widely promoted as an approach to mitigating climate change despite limited data on carbon removal pathways and uncertainty around benefits and risks at operational scales. We explored the feasibility of climate change mitigation from seaweed farming by constructing five scenarios spanning a range of industry development in coastal British Columbia, Canada, a temperate region identified as highly suitable for seaweed farming. Depending on growth rates and the fate of farmed seaweed, our scenarios sequestered or avoided between 0.20 and 8.2 Tg CO_2_e year^−1^, equivalent to 0.3% and 13% of annual greenhouse gas emissions in BC, respectively. Realisation of climate benefits required seaweed-based products to replace existing, more emissions-intensive products, as marine sequestration was relatively inefficient. Such products were also key to reducing the monetary cost of climate benefits, with product values exceeding production costs in only one of the scenarios we examined. However, model estimates have large uncertainties dominated by seaweed production and emissions avoided, making these key priorities for future research. Our results show that seaweed farming could make an economically feasible contribute to Canada’s climate goals if markets for value-added seaweed based products are developed. Moreover, our model demonstrates the possibility for farmers, regulators, and researchers to accurately quantify the climate benefits of seaweed farming in their regional contexts.

## Introduction

Climate change, driven largely by increasing atmospheric carbon dioxide (CO_2_), is now one of the greatest challenges threatening humanity and global ecosystems^1,2^. Carbon dioxide removal (CDR) strategies are increasingly seen as necessary for meeting global climate targets^3^, with seaweed aquaculture recently gaining attention as a promising approach^4–6^. This interest in seaweed is due to the high productivity of many species and their efficiency at drawing CO_2_ from the water and converting it into organic biomass^7^. Oceans also play a significant, natural role as a carbon sink, taking up an estimated 2.8 Gt C year^−1^ in the 2011–2020 period, equivalent to approximately 30% of annual fossil fuel emissions^8^. As such, several strategies have emerged to try to enhance the rate of carbon sequestration and storage in the ocean by protecting, restoring, or enhancing productivity of wild marine plants, macroalgae, and phytoplankton^6,9,10^.

Seaweed farming currently accounts for over 50% of global marine and coastal aquaculture production by weight^11,12^ with the vast majority (over 99%) currently grown in Asia^13,14^. Most farmed seaweed is used as food, either consumed directly or as a food additive^13,15^, but in some economies (i.e., South Korea) seaweeds are increasingly being funneled into secondary production of abalone, also for human consumption^16^. Recent estimates have suggested that between 48 and 119 million km^2^ of the global ocean (an area 24–60 times the size of Greenland) may be suitable for seaweed production^5,17^, however the industry remains nascent in most countries^5,18^.

Recently, large-scale farming of seaweed has been put forward as a potential CDR strategy, with various groups including the International Panel on Climate Change highlighting seaweed aquaculture as an important area for research and development^4,19–21^. In response, a variety of approaches to farming seaweed for the express purpose of CDR have been proposed, including the purposeful transport of seaweed biomass to the deep ocean where it can remain for long periods^22–25^, and the use of seaweed to produce lower emission products such as biofuel^26,27^. Such approaches remain largely untested, and their efficacy and ecological impacts remain uncertain^6,14,28^.

Much of the net primary production of wild seaweed, over 80% by some estimates^29^, is released as detrital particulate and dissolved organic carbon (POC and DOC)^30,31^. Most of this POC and DOC is consumed and recycled, nourishing coastal ecosystems and driving secondary production^32,33^. Unconsumed, recalcitrant POC and DOC can be transported to nearby sediments or exported by currents into deeper water where it is sequestered^34–36^. Unlike wild seaweeds, farmed seaweed is typically harvested after the growing season, likely reducing the POC and DOC produced^37^. While, recent proposals to actively sink seaweed, either around farms or in deep waters, aim to facilitate and enhance this natural sequestration process^5,38^, industrial-scale sinking of seaweeds is increasingly seen as ecologically risky and a socially irresponsible use of biomass^39^.

Beyond passive sequestration and active sinking, harvested seaweed biomass may help reduce atmospheric greenhouse gas emissions if used as a replacement for products that use more land, water, and carbon resources. Such products include foods and food additives, animal feed, biofuels, soil additives (biochar and biostimulants), and pharmaceuticals and cosmetics^40–42^. Seaweed aquaculture typically requires minimal inputs of material and energy, uses no fertilizer, and produces limited emissions and can therefore help decarbonize production systems by replacing carbon-intensive alternatives^43–45^. A large proportion of global seaweed production, approximately 31–38%^15^, is currently used for direct human consumption and there is substantial interest in using seaweed to create more sustainable food systems and supply chains^44,45^. Other uses of seaweed such as cement additives or biochar for agriculture^42^ can directly sequester carbon in built environments and soils. However, the ability for seaweed products to reduce emissions by replacing higher intensity products depends critically on the emissions profile of the food or product that seaweed is replacing, the efficiency of seaweed production and processing, and a sufficient market for the seaweed products.

Farming seaweeds also has other potential benefits including reducing excess anthropogenic nutrients in ocean water, nourishing coastal ecosystems, and reducing wave impacts on shorelines^4,46,47^. Due to the potential for carbon reduction and these co-benefits, there is considerable excitement around seaweed aquaculture and substantial marketing, investment, and media attention focused on the climate and ecosystem benefits of the industry^24,48^. Given this enthusiasm, there is an urgent need to ensure our understanding of the benefits as well as risks of seaweed aquaculture informs potential industry development^14,49^.

Mathematical models provide a valuable tool for gaining insight into this emerging industry and its potential climate benefits. To date, models of seaweed aquaculture have largely been at the global scale, investigating the extent of suitable area^5,17,50,51^, or its potential as a CO_2_ removal strategy^5,17,52,53^. However, global models have significant uncertainties when examined locally, where local and regional environmental and social factors are key to determining the benefits and risks of seaweed aquaculture. Conversely, local seaweed aquaculture models have largely focused on the economics of seaweed production and processing^54–56^ or on life cycle assessments of a specific farm system^26,41,57^ yielding results that are difficult to generalize.

In this study, we endeavor to bridge this divide between local and global models by assessing the climate change mitigation potential of seaweed aquaculture at the regional scale, within a specific jurisdiction. We target knowledge gaps around the efficacy of sequestration pathways and the associated emissions using a place-based, data-driven, mathematical model. We consider several CO_2_ sequestration and emissions pathways focusing on seaweed production and processing emissions, and marine sequestration. We also conduct a preliminary assessment of economic feasibility. We applied our model to a case study in Canada, a nation identified to have extensive—but unexplored—potential to develop seaweed aquaculture for climate mitigation purposes^18,58^.

We based our model on a case study of kelp (seaweeds of order Laminariales) aquaculture in British Columbia (BC)—a province with a long coastline of nutrient rich waters, abundant wild seaweeds suitable for cultivation, and an expanding kelp farming industry. Using a suite of scenarios representing increasingly ambitious aquaculture development and technological advances, we estimated a range of net annual atmospheric draw-down and avoided emissions values (Tg CO_2_e year^−1^). We grounded our model in discussions with regional kelp producers and production estimates from local kelp aquaculture operations and the published literature (See “Methods” section and “Supplementary Material”).

## Results

**Figure 1:**
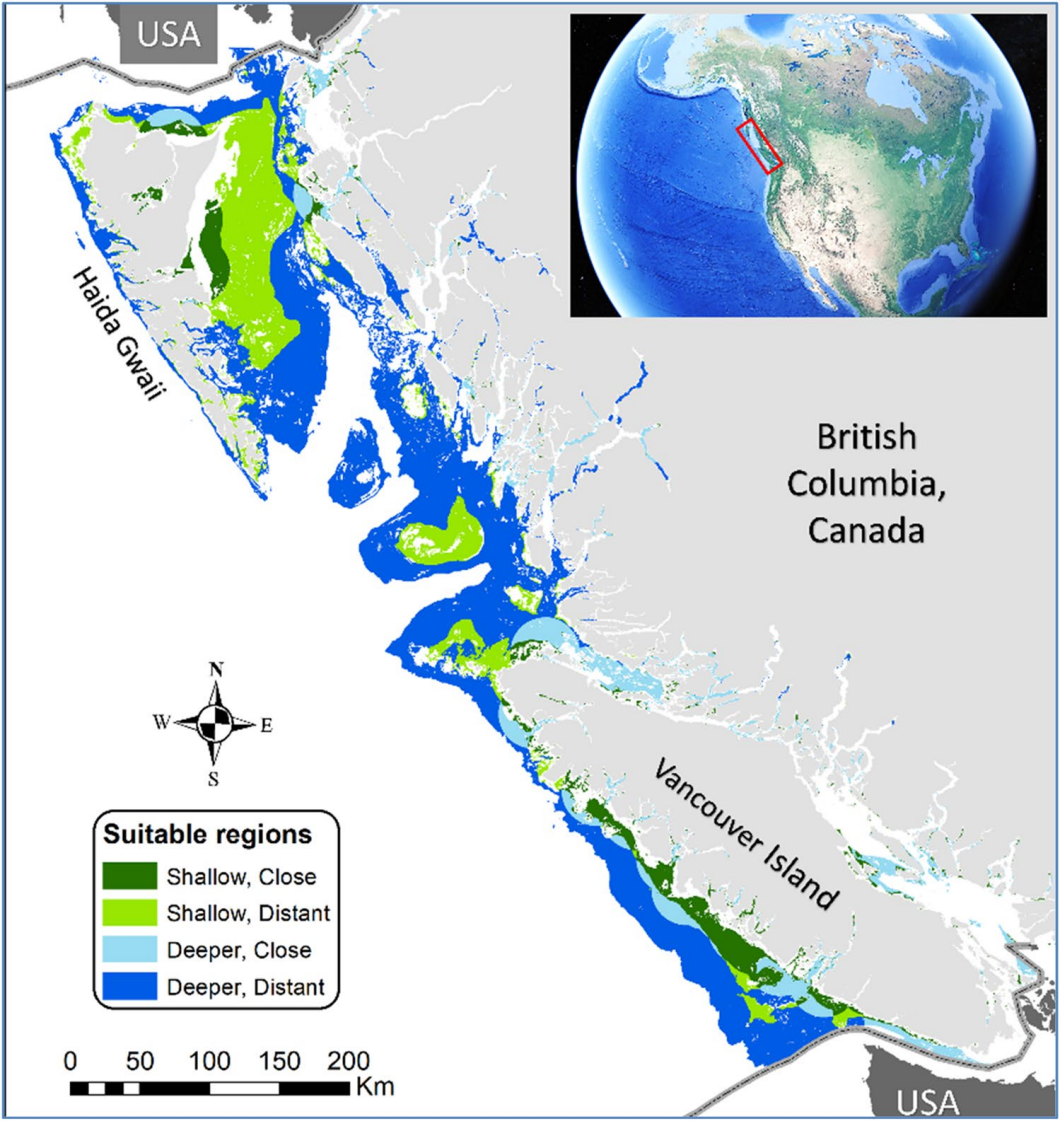
Potentially suitable areas for seaweed aquaculture in BC, Canada. Our study area showing suitable locations cultivated under different production scenarios (main panel) and the location of our study in North America (inset). We defined suitable areas as those with soft substrates, at optimal depths (≥ 15 and ≤ 200 m), with low human use. Shallow suitable waters close to communities (dark green) are assumed to be favored under the Local scenarios; the areas available for cultivation under the *Expanded* scenarios also include deeper waters close to communities (light blue). The *Techno Industrial* scenario includes cultivation across all suitable areas. Small pockets of shallow areas close to communities on the eastern side of Vancouver Island, as well as deeper locations in the mainland inlets are notable for early kelp farm development. See the “Methods” for details on the scenarios and our methods for identifying suitable areas.

We examined five scenarios reflecting a range of potential kelp aquaculture futures in BC (Table 1). For each scenario we calculated the spatial extent of potential kelp farms using assumptions about suitability (depth and substrate), access to coastal communities and infrastructure, and overlap with other human uses (Fig. 1). The scenarios varied in the spatial extent of kelp aquaculture, kelp production rates, and the fate of farmed biomass (Table 1).

**Table 1:** Summary of the five scenarios examined.

Scenario (horizon)	Description	Area (km^2^)	Kelp production (kg ww m^−2^)	Kelp biomass fate
Local-No harvest	In depths from 15 to 75 m with suitable substrate; within 25 km of a coastal community and avoids crowded spaces All kelp grown is left/released at site, no kelp transport or processing	507	Average reported by regional farmers: Sacch: 0.78 Alaria: 0.22 Nereo: 0.26	100% near farm passive release
Local-products	Same footprint as ‘No Harvest’, majority of biomass is harvested to produce products			10% near farm passive release 80% food 10% animal feeds
Expanded	Industry expanded to deeper water but still close to communities. Product mix reflects evolved demand	1210		10% near farm passive release 60% food 20% animal feeds 10% biofuel
Expanded-optimized	Same footprint as ‘Expanded’ but production rate increased, assuming optimization (e.g., harvest timing, strain selection)		Average of published values used for all species: 8.3	
Techno industrial	Farms expanded to suitable depths across the coast. Market saturation leads to active sinking	5681		50% active sinking 30% food 10% animal feeds 10% biofuel

For each scenario, the size of the growing area, the mean rate of kelp production, and the fate of kelp biomass is outlined All scenarios use a species mix (80% *Saccharina latissima* (Sacch), 10% *Alaria marginata* (Alaria), and 10% *Nereocystis luetkeana* (Nereo)) reflecting the current product mix in BC.

### Net climate benefits

Our simulations indicate that kelp aquaculture has the potential to provide substantial climate benefits, although there is large variability in model estimates. We present our results for each model scenario as the median value from 10,000 Monte Carlo runs, along with the 25th and 75th percentiles. For consistency, we present all CO_2_e results in units of Tg (i.e., 10^12^ g, or 1 million metric tonnes).

**Figure 2:**
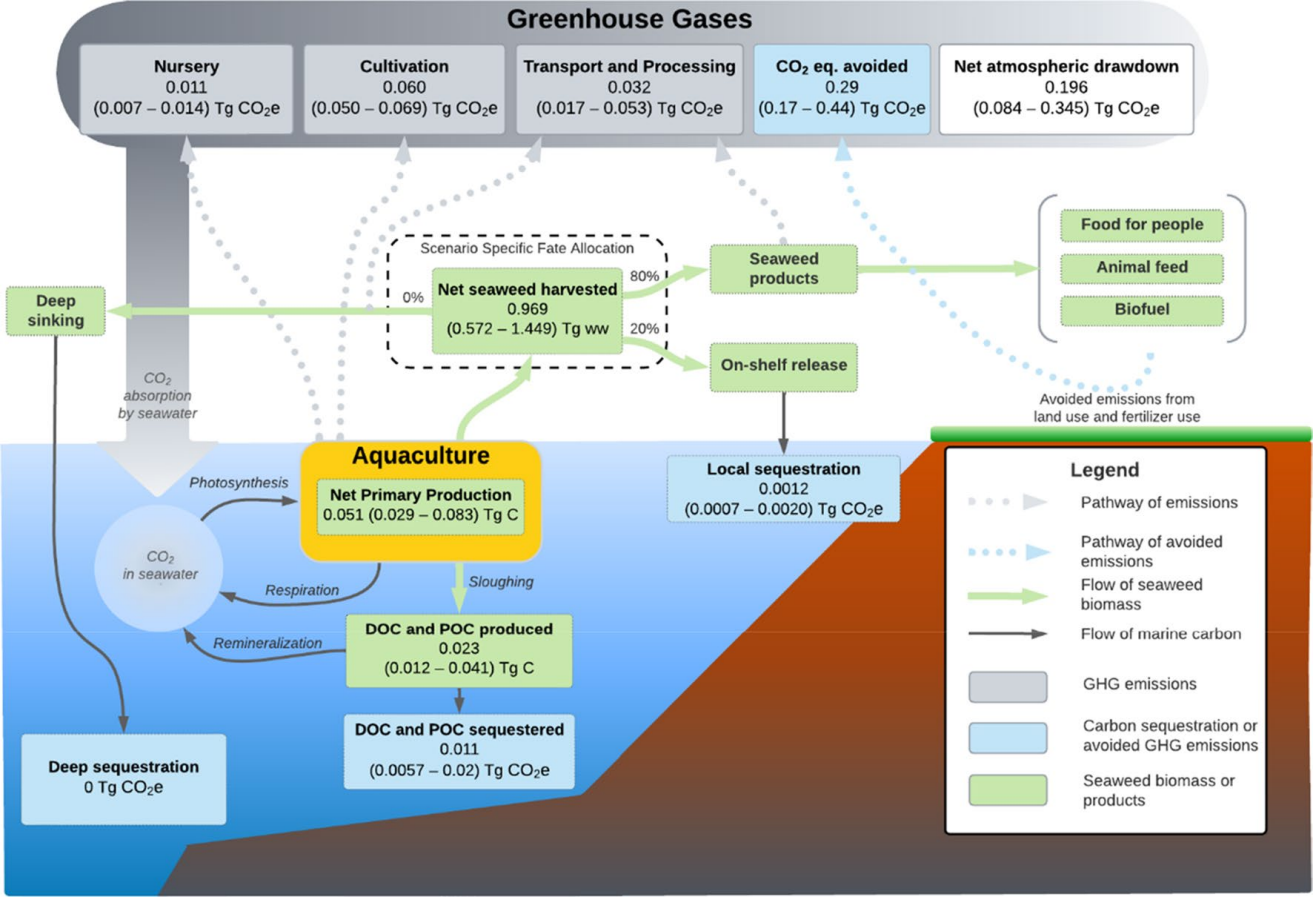
Illustrative model structure. Diagram illustrating the structure and carbon pathways represented in the mathematical model. Results for key model elements are shown for the *Expanded* scenario as median (25th percentile–75th percentile) estimates from 10,000 Monte Carlo runs. Note that median values from Monte Carlo simulations do no sum precisely. Results for all scenarios are provided in Table S1. Processing emissions not considered include product packaging, storage, and transport and other unquantified life cycle components (see “Supplementary Material”).

As an example of an intermediate level of industry development, our *Expanded* scenario (Fig. 2) estimated a net atmospheric reduction of 0.196 (0.084–0.345) Tg CO_2_e year^−1^ (results for other scenarios are provided in Table S1). This climate benefit is achieved by producing 0.969 (0.572–1.45) Tg ww of harvestable kelp per year, directing 80% of it to seaweed-based products (primarily food and animal feed), and leaving 20% of it in the water where it may be consumed, re-mineralized, and partially sequestered. In this scenario, seaweed products avoid the release of 0.29 (0.17–0.44) Tg CO_2_e year^−1^ by replacing existing, more carbon-intensive products. The biomass intentionally left in the water sequesters only 0.0012 (0.0007–0.002) Tg CO_2_e year^−1^, about 200 times less. During the growing period, from an estimated loss of 0.023 (0.012–0.041) Tg of carbon as POC and DOC we estimate about half (0.011, 0.0057–0.020 Tg CO_2_e year^−1^) would be sequestered. We predicted nursery operations would emit 0.011 (0.007–0.014) Tg CO_2_e year^−1^; at-sea cultivation operations to emit 0.060 (0.050–0.070) Tg CO_2_e year^−1^; and transport and processing of harvested seaweed to emit 0.032 (0.017–0.053) Tg CO_2_e year^−1^.

**Figure 3:**
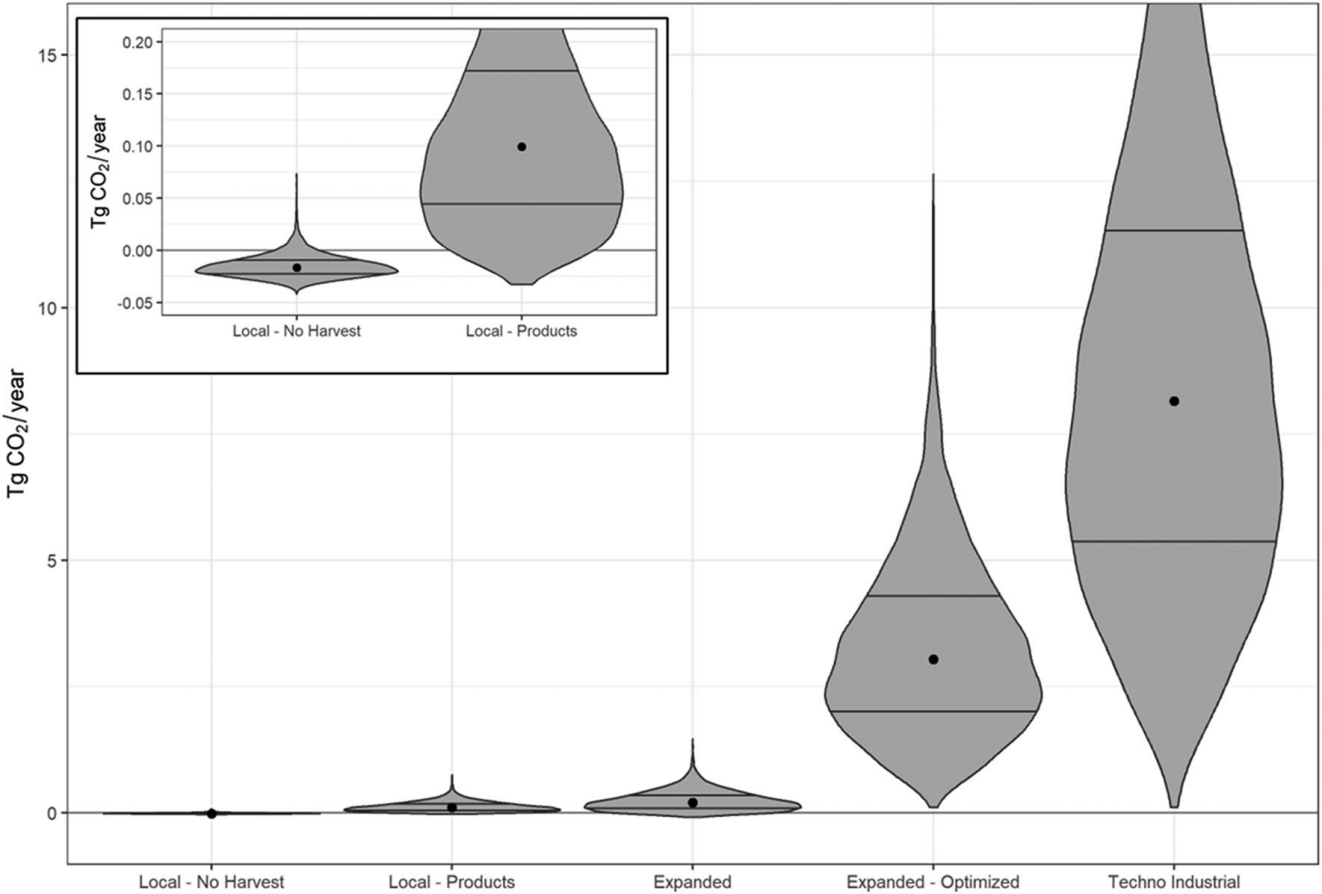
Net reduction in atmospheric CO_2_ for each scenario. Violin plots illustrate the distribution of estimates from 10,000 Monte Carlo runs, with the central dot indicating the median and horizontal lines at the 25th and 75th percentiles. Inset plot shows a re-scaled version of the two *Local* scenario results.

A key result from our analysis is that seaweed-based products have substantially more potential for reducing atmospheric greenhouse gas emissions through product replacement than by sinking seaweed for carbon sequestration. This holds true for all five scenarios, and is emphasised by our *Local-No Harvest* scenario which suggests that leaving all farmed kelp biomass in the water may generate more CO_2_ than would be sequestered (Fig. 3). Actively sinking kelp in deep water (e.g., the *Techno Industrial* scenario) results in almost four times more sequestration than near-farm release but remains much less effective than the production of kelp-based replacement products (Supplementary Table S2). Thus, our results suggest marine carbon sequestration alone is unlikely to justify kelp aquaculture as a CDR strategy.

### Economic assessment

We estimated the monetary costs of seaweed aquaculture and the potential value of seaweed-based products associated with each of the five scenarios to provide insight into the economic feasibility of this industry in BC. The estimated annual total cost of the seaweed aquaculture scenarios (including nursery and farm operation, transport, and processing) ranged from $183.58 (175.16–195.1) million CAD in the *Local-No Harvest* scenario to $3028.44 (2694.28–3439.19) million CAD in the *Techno Industrial* scenario, while the value of seaweed products was highest in the *Techno Industrial* scenario at $2302.91(1606.39–3093.16) million CAD (Table 2). The only scenario in which product value exceed costs was the *Expanded-Optimized* scenario, which benefits from increased seaweed productivity and a large proportion of the seaweed being directed to higher-value food products. This scenario yielded a profit of $347.63 (108.01–616.33) million CAD.

**Table 2:** Estimated annual total costs and product values for each scenario in Canadian dollars.

Scenario	Total cost (CAD $M)	Product value (CAD $M)	Overall net cost (CAD $M)	Net Cost per Tg CO_2_e (CAD $M)
Local*-*No Harvest	$183.58 (175.16–195.1)	$0	$183.58 (175.16–195.1)	NA
Local*-*Products	$188.17 (179.51–199.98)	$35.36 (21.17–52.87)	$153.21 (136.11–168.48)	$1339.22 (700.94–2857.78)
Expanded	$449.09 (428.41–477.26)	$78.61 (47.17–116.88)	$371.43 (333.24–405.99)	$1539.42 (793.83–3296.81)
Expanded*-*Optimized	$554.12 (512.3–601.89)	$902.89 (630.66–1213.74)	− $347.63 (− 616.33 to − 108.01)	− $105.5 (− 152.14 to − 47.02)
Techno Industrial	$3028.44 (2694.28–3439.19)	$2302.91 (1606.39–3093.16)	$795.1 (294.43–1211.91)	$93.84 (26.33–210.72)

For each of the five scenarios examined, the median estimate in millions of Canadian dollars (CAD $M), followed by 25th and 75th percentile in parentheses, is provided for the total cost, the value of seaweed replacement products, the net cost, and the net cost per Tg CO_2_e sequestered or avoided. Total cost includes the cost of production, transport, and processing.

When costs are calculated relative to the estimated carbon sequestered or avoided, to examine the cost to provide this natural climate solution, we find a substantial differences between scenarios. The cost of climate benefits in the *Local-Products* and *Expanded* scenarios are high at $1339 (701–2858) million CAD Tg CO_2_e^−1^ and $1539 (794–3297) million CAD Tg CO_2_e^−1^, respectively. Net cost is reduced in the expansive *Techno Industrial* scenario to $94 (26–211) million CAD Tg CO_2_e^−1^, and even becomes profitable in the *Expanded-Optimized* scenario with an estimated profit of $106 (47–152) million CAD Tg CO_2_e^−1^. This variability in the costs of achieving climate benefits is driven both by increased product values and climate benefits in across the scenarios (see following section).

### Differences between scenarios

Model estimates of climate benefits varied by scenario due to differences in spatial extent, production rates, and the fate of harvested kelp (Fig. 3). Under our most conservative scenario (*Local-No Harvest*) kelp aquaculture was a net emitter of CO_2_, producing 0.02 (0.010–0.023) Tg CO_2_e year^−1^ and suggesting total emissions from production would exceed this scenario’s sequestration potential (Supplementary Figs. S1 and S2, Table S1). In contrast, our most developed scenario (*Techno Industrial*) resulted in a net draw down of 8.15 (5.39–11.53) Tg CO_2_e year^−1^. This was primarily comprised of emissions avoided by seaweed*-*based products (8.9 [6.0–12.3] Tg CO_2_e year^−1^) and to a lesser extent biomass sequestered via active sinking in deep water (0.71 [0.44–1.10] Tg CO_2_e year^−1^ (Supplementary Fig. S1 and Table S1). Total emissions for this scenario were estimated at 1.40 (0.99–1.92) Tg CO_2_e year^−1^. The *Local-Products* scenario is similar to the *Expanded* scenario, while the *Expanded-Optimized* scenario falls in between the *Expanded* and *Techno Industrial* scenarios. The *Local-Products* scenario estimates a net reduction of 0.10 (0.045–0.172) Tg CO_2_e year^−1^ while the *Expanded-Optimized* scenario yields a net reduction of 3.04 (2.02–4.29) Tg CO_2_e year^−1^. Uncertainties are high and the range of plausible values is large for all model scenarios.

### Sensitivity analysis

We applied two sensitivity analyses to examine uncertainty in the model. We first assessed the influence of uncertainty at the scale of the three main sub-models (production, emissions, and products-sequestration). The second analysis examined the sensitivity of model results to uncertainty in the individual model parameters within each sub-model. These results are scenario-specific as they depend on the scenario configurations (results for all scenarios are provided in Supplementary Figs. S3–S4).

**Figure 4:**
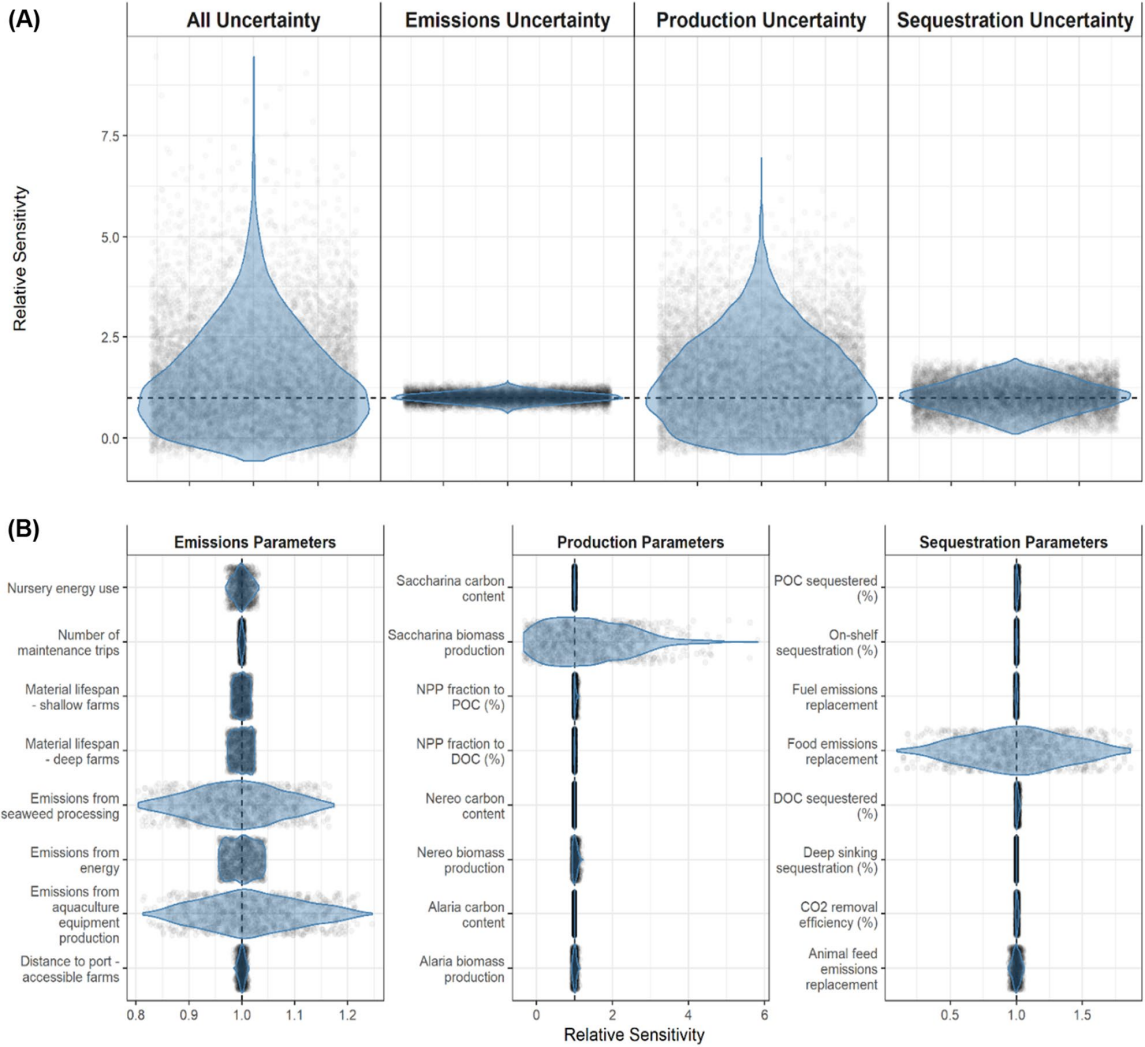
Relative sensitivity of the estimated net reduction in atmospheric CO_2_. Results are shown for the *Expanded* scenario, as (**A**) cumulative uncertainty in different parameter categories, and (**B**) uncertainty in individual parameters. Sensitivity is assessed relative to a model run with no uncertainty, where model parameters were set to their central estimate, yielding a net CO_2_ reduction of 0.155 Tg CO_2_ year^−1^. Violin plots show the distribution of the Monte Carlo runs, with each individual estimate shown as a shaded point. Note the plots in panel (**B**) have different x-axis scales.

Of the three sub-models, uncertainty in production contributed most to the overall model uncertainty (Fig. 4A; *Expanded* scenario). Uncertainty in sequestration parameters contributed a smaller amount while emission parameters contribute the least to overall model uncertainty. The parameter-level sensitivity analysis identified the parameters with the highest uncertainty in each sub-model (Fig. 4B). Species-specific production rates contribute the most uncertainty to the production sub-model, most obviously with *Saccharina* as it's the dominant species farmed. In some iterations, variability in the production rate of *Saccharina* can result in more than a fourfold increase in the estimated net CO_2_ reduction (Fig. 4B). In the sequestration sub-model the food emission replacement factor had the highest uncertainty, with estimates of net CO_2_ reduction varying by more than ± 50% depending on the value of this parameter. Parameters estimating material production (concrete, steel, etc.), energy use, and seaweed processing contributed the most uncertainty to the emissions sub-model. However, this sub-model contributed little to the overall model uncertainty.

Our modelling shows a wide range of plausible results within scenarios, as well as significant differences between them. The former reflects uncertainty in the model parameters, while the latter reflects the uncertain future of seaweed aquaculture development in BC. Exploration of uncertainty using sensitivity analyses identified kelp production rates as a key source of uncertainty. The estimated production rates provided by regional seaweed producers were variable, as well as substantially lower than some global estimates, which resulted in large uncertainties for these model parameters (i.e., a mean of 0.78 kg ww m^−2^ for *Saccharina latissima* vs. an average literature value of 8.3 kg ww m^−2^, see “Supplementary Methods” for further information). Production rates in line with average values from the literature were used in two of our scenarios (*Expanded-Optimized* and *Techno Industrial*) to reflect the potential for increased production as the industry in BC develops.

Although less significant than production rates, model estimates of net climate benefit are also sensitive to numerous other sources of uncertainty (e.g., the emissions offset by replacing conventional food products with a seaweed-based alternative, the emissions from production of material used in aquaculture operations, the export of seaweed carbon from aquaculture, and the flux of CO_2_ from the air to surface waters)^28^. Improving our understanding of these various processes, particularly as they apply to local settings, will be critical for accurately quantifying the climate benefits of seaweed aquaculture.

## Discussion

In this analysis we explored a range of seaweed aquaculture development scenarios and various pathways for sequestering carbon or avoiding CO_2_ emissions. Our results indicate that, while not a silver bullet, seaweed aquaculture could reduce greenhouse gas concentrations, particularly if harvested biomass is used to create products that can replace traditional, higher-emission products. Our coarse economic analysis illustrates the importance of high-value seaweed products (i.e., food, biofuel, and animal feed) for improving the feasibility of seaweed aquaculture as a natural climate solution. By grounding the model in the local context of BC, we also provide, for the first time, an estimate of the potential climate benefits from kelp aquaculture in Pacific Canada. Our sensitivity analyses highlight where targeted future research would improve our understanding of seaweed aquaculture systems.

Our model illustrates that marine sequestration pathways are likely to sequester only a small fraction of seaweed biomass because of high rates of re-mineralization, inefficient transport to depth, and variability in how much the carbon in seaweed biomass contributes to atmospheric drawdown^28,59^. In comparison, if seaweed-based products can replace existing products that have high, well-described emissions, significant reductions in atmospheric greenhouse gases could be achieved. Specifically, we estimate that with optimized production seaweed farmed to replace traditional food products (e.g., oil crops, pulses, or cereals) could avoid 0.00324 (0.00209–0.00470) Tg CO_2_e km^−2^ year^−1^. Alternatively, the intentional sinking of seaweed biomass in deep water would sequester less than 1/10th of this amount. These estimates show good concordance with values from the literature: one recent study^17^ found a similar magnitude of potential benefits for food replacement pathways, while sequestration in deep water has previously been estimated to sequester between 0.00006 Tg CO_2_e km^−2^ year^−1^ ^38^ and 0.00111 Tg CO_2_e km^−2^ year^−1^ ^5^.

With an appropriate mix of seaweed fates, our results show that modest development scenarios (*Local-Products* and *Expanded*) reduce atmospheric greenhouse gases by 0.10 and 0.20 Tg CO_2_e year^−1^, respectively. These reductions correspond to 0.15–0.3% of total annual emissions from BC (64.6 Tg CO_2_e year^−1^ in 2020^60,61^). Our most ambitious scenario (*Techno Industrial*) led to a reduction of 8.2 Tg CO_2_e year^−1^, or 12.6% of BC’s annual emissions. This substantial range of possibilities shows that reducing or offsetting society-wide emissions with kelp aquaculture must be weighed against other, more localised costs and benefits. Given the assorted non-monetary benefits and risks from seaweed aquaculture (discussed below), locations which maximize the benefits and manage the risks to provide win–win scenarios are likely to be most successful. This could include co-locating seaweed and shellfish in multi-trophic aquaculture, or locating seaweed farms in areas of high anthropogenic nutrient input^62–64^.

The economic analysis provides additional insight into the feasibility of seaweed aquaculture in BC. We find the net cost for every Tg CO_2_e sequestered or avoided varies substantially between scenarios, with low cost or even profit achieved in our most ambitious scenarios. The economic feasibility of these scenarios is a function of increased kelp production rates and the large proportion of harvested biomass used for replacement products. These factors will thus influence the economic viability of kelp aquaculture. However, this analysis provides only a preliminary insight into the economics of kelp aquaculture in BC, because a lack of local data required the use of cost and product value data from other regions or global models. The local context of seaweed farms will influence both the cost of seaweed production, processing, and the manufacturing and market for replacement products. It will also determine the potentially significant non-monetary benefits. Quantifying local social and economic costs and benefits is therefore necessary to refine such estimates.

Our results indicate that the climate benefits of seaweed-based products are largely dependent on their ability to replace products with higher associated emissions. Here we have assumed robust markets for kelp-based food, biofuel, and animal feed, however market growth and the development of markets for novel products will play a key role in the success of this industry. While various markets currently exist, key factors in their success include the availability and cost of seaweed biomass, increased consumer awareness and demand for bio-based products, and government initiatives to promote green products^65^. The appropriate allocation of carbon credits could also provide further incentives for seaweed farming and seaweed-based products^28,58^.

Most of the world’s seaweed aquaculture is currently consumed as food, near where it is produced in East Asia^66^. While our results indicate the best outcome for CO_2_ reduction would be the adoption of seaweed-based foods by the developed North, the barriers to dietary change are notoriously difficult to overcome^67^ and the processing and regulatory requirements for human consumption^66^ are likely to be the highest of any kelp-based products. Nevertheless, existing markets for human consumption of seaweed provide some reason for optimism, and this market could be expanded by marketing seaweed-based food products for their considerable health benefits^49,66^ and low environmental footprint^18,68^. Novel foods and processing methods, such as plant-based meats, may also provide opportunities for seaweed to more easily be adopted into global diets.

Our model indicates the production of biofuels from seaweed offers another avenue for direct carbon reduction, and this outcome may encounter fewer regulatory and societal barriers than human consumption. A recent review of seaweed-based biofuel shows that since 2000, European countries have invested significantly in seaweed farming for biofuel and biochemical production^65^. Current market trends suggest that mixed alcohol production could be economically competitive^65^, while integrated biorefineries can recover over 90% of the initial biomass as 6 different products with high market values^69^. However, biofuels and biorefineries are among the most capital intensive seaweed product pathways, requiring long term policies and reliable biomass production.

Atmospheric greenhouse gases can also be reduced by incorporating seaweed into animal feeds. Seaweed has been identified as a promising alternative to corn and soybean in animal feeds, not only providing climate and land-use benefits but in some cases also improving animal growth and meat quality^70^. More nascent products, such as methane-reducing cattle feed additives have also shown promise^17,71^, but face greater uncertainty and regulatory hurdles^72^ and were not considered in our model. In the agriculture sector more broadly, seaweeds have a long history of use as a bio-fertilizer in coastal areas all over the world^73^. Their eco-friendly properties make them increasingly important for sustainable agriculture in organic and integrated organic farming^73^, providing another agricultural market for seaweed biomass.

The feasibility of seaweed aquaculture and the resulting products would benefit from reducing the uncertainties in our estimates of carbon emissions avoided. Sensitivity analyses show our estimates of climate mitigation potential are highly sensitive to the amount of seaweed that can be produced and the emissions that can be avoided by seaweed-based products. Addressing these uncertainties will require further research on seaweed production rates as well as the downstream emissions associated with both seaweed-based products and the traditional products they seek to replace. Optimization of seaweed production rates will need to consider variability in rates of growth and erosion, ocean climate, nutrient limitation, and timing of crop harvest^37,74^, none of which were able to be considered here. Similarly, the values available to parameterise the emissions offset by seaweed replacement products are limited to emission reductions due to land use and nutrient management and do not account for additional associated emissions from traditional products (e.g., those related to fertilizer production, agricultural energy use, and product storage and transport). Optimizing production rates, a broader consideration of emissions related to both seaweed and traditional (potentially replaceable) products, and accounting for sources of uncertainty will provide greater insight into the climate benefits of seaweed-based products, further enhancing their market appeal.

Our predicted estimates of CO_2_ reduction by kelp aquaculture in BC compare favourably to recent estimates for natural climate solutions across Canada^75^. Specifically, estimates from our *Local-Products* scenario are similar to strategies such as seagrass restoration (0.1 Tg CO_2_e year^−1^ in 2050), while our maximalist *Techno Industrial* scenario compares to more substantial strategies such as improved agricultural nutrient management or the use of cover crops (6.3 and 9.8 Tg CO_2_e year^−1^ in 2050, respectively)^75^. When considering the cost of this climate mitigation potential, kelp aquaculture in the *Expanded-Optimized* and *Techno Industrial* scenario are competitive with other natural climate solutions across Canada and fall below the upper price point (the anticipated cost of one Tg CO_2_e by 2030) set by Drever et al. of $100 million CAD^75^. In particular, the potential profitability of seaweed aquaculture in the *Expanded-Optimized* scenario suggests that it may, in some contexts, be a highly desirable climate solution. However, the median estimated cost of carbon reduction in other, less productive scenarios exceeds $1000 million CAD Tg CO_2_e^−1^, far more than the cost of other natural climate solutions offering similar or greater climate mitigation potential^75^. An additional benefit of seaweed aquaculture is that while it may conflict with other uses of the marine environment, it does not conflict with other land uses such as food and bioenergy production as many terrestrial natural climate solutions do^75,76^. It may thus facilitate terrestrial solutions by making space for land based conservation, restoration, and regenerative land management^17^.

Beyond the potential climate benefits and economic feasibility of kelp aquaculture, its value also needs to consider a number of societal and ecological questions. Seaweed aquaculture has been shown to provide sustainable livelihoods and contribute to community well-being in some local and regional contexts^46,64,77^. In BC, seaweed aquaculture expansion would likely require development in remote coastal communities, as transport costs will likely necessitate pre-processing such as drying to be done close to where kelp is harvested. As most of these communities are within the territories of Indigenous people and governments, partnering with Indigenous communities will be essential to ensure the growing industry and its supply chains will benefit, rather than impact, Indigenous waters, communities, and rights. This will require a just regulatory environment to ensure development proceeds in an equitable, rights_-_driven manner^58,78,79^. There is also potential for conflict between seaweed aquaculture and other marine industries (e.g., tourism, shipping, fisheries, and other types of aquaculture) if the industry should develop to the extent envisioned by our more ambitious scenarios. Navigating these conflicts, possibly through marine spatial planning, will be essential^80,81^.

Ecologically, the potential positive and negative effects of expansive seaweed aquaculture are myriad. Seaweed aquaculture can improve water quality^62,82^, protect shorelines^4,83^, create refugia from ocean acidification^4,84^, and provide habitat and nutrients to various marine species^82,85–87^. On the other hand, the large areas required for effective CDR may lead to competition for nutrients and light, reducing productivity of wild seaweeds, phytoplankton, and benthic communities^6,53,59^. Further complicating matters, these impacts and benefits are likely to be context dependent^46^, with site and species being critical considerations^88^. There are also risks of harmful algal blooms, non-native species introductions, endemic and emerging pathogens and diseases, and the potential for genetic interactions with wild seaweed populations^46,89,90^. Marine sequestration of seaweed biomass may also negatively impact nearby sediments^46,91^, as well as mesopelagic and deep-sea food webs and water chemistry^6,38,92^. Further research into these and other social, economic and ecological implications will be critical for guiding the development of seaweed aquaculture in Canada, and elsewhere around the world.

Climate change caused by increasing atmospheric CO_2_ concentrations is a global challenge, however kelp aquaculture is a place-based CDR strategy, making assessments based on national or global averages of limited use to those on the ground. This means that the success of CDR strategies will depend very much on local environmental and ecological suitability, as well as local production costs and supply chains, community buy-in, and good governance^79^. We therefore advise caution in relying on global models and parameters^5,34,52,93^ to assess local feasibility, as these are unlikely to provide the accuracy necessary to answer essential ecological, economic, and certification questions. To advance assessments of feasibility, we need models parameterised for local social-ecological contexts.

By integrating seaweed aquaculture industry emissions, in-water sequestration, and emissions avoided by seaweed-based replacement products, this work provides novel insight into the potential role of seaweed aquaculture as a climate change mitigation strategy. Despite the significant uncertainties, our results indicate that kelp aquaculture can contribute to atmospheric greenhouse gas reduction, with values for BC on par with other natural climate solutions examined across Canada, thus helping BC and Canada achieve their climate goals. Realising this potential will require replacing carbon-intensive products with lower emission, seaweed-based products, and a robust market for these products. Marine sequestration pathways are important to consider, but appear unlikely to have substantial climate benefit and entail potentially significant environmental consequences. Further research to refine our understanding of the pathways modelled here will help advance our understanding of the potential climate mitigation benefits of seaweed aquaculture.

## Methods

### Experimental design

The mathematical model developed here aims to describe the carbon sequestration potential as well as the associated emissions for seaweed aquaculture and various possible fates for harvested seaweed biomass. The structure of the model was informed by reviewing several published seaweed aquaculture models^6,38,45,52,54^, and consists of 12 equations with a total of 81 parameters. We divided the model into sub-models for seaweed production, carbon sequestration and product fates, and emissions. The production sub-model first estimates the biomass of seaweed that could be produced in the defined study area. We then track the fate of this biomass along the pathways represented in the carbon sequestration and products fate sub-model, and estimate the emissions produced along each pathway with the emissions sub-model. We used the R statical package^94^ to develop our model. Here, we outline the main equations and briefly describe how each was parameterised. Additional details are provided in the “Supplementary Methods”.

#### Seaweed production

We calculated the species-specific biomass of kelp produced across the study area as:

1 $${{B}_{H}}_{i}={{B}_{A}}_{i}*A*{S}_{i}*{H}_{N}$$

where ${{B}_{H}}_{i}$ is the total wet weight (ww) of biomass harvested annually for species $i$ (kg ww year^−1^); ${{B}_{A}}_{i}$ is the area-based kelp harvest for species $i$ (kg ww m^−2^); $A$ is the total area used for kelp production (m^2^); ${S}_{i}$ is the proportion (unitless) of the area used to produce species $i$; and ${H}_{N}$ is the number of harvests per year. We included the three kelp species most commonly farmed in BC: *Saccharina latissima* (sugar kelp), *Alaria marginata* (ribbon/winged kelp), and *Nereocystis luetkeana* (bull kelp)*.* The model does not explicitly account for nutrient availability or other factors that may influence production rates. Instead, we used a range of seaweed production estimates obtained from local seaweed producers and the literature to capture the variability in production rates across different environments.

Some portion of kelp biomass produced is lost as detritus prior to harvest in the form of POC and DOC^29,34^. While likely small compared to harvested biomass, this POC and DOC can contribute to sequestration. We therefore related POC and DOC portions to ${B}_{H}$ by first back-calculating the net primary productivity (NPP) as:

2 $$NPP=\frac{\sum_{i=1}^{i}{{B}_{H}}_{i}*{DW}_{i}*{C}_{i}}{1-({FL}_{DOC}+{FL}_{POC})}$$

where $NPP$ is in (kg C year^−1^); ${{B}_{H}}_{i}$ is the total harvested biomass for species $i$ (kg ww year^−1^); ${DW}_{i}$ is the wet- to dry-weight conversion for species $i$ (kg dw kg ww^−1^); ${C}_{i}$ is the carbon content of species $i$ (kg C kg dw^−1^); ${FL}_{DOC}$ is the estimated fraction of kelp carbon lost as DOC (unitless); and ${FL}_{POC}$ is the estimated fraction of kelp carbon lost as POC (unitless).

With NPP estimated, we then calculated POC and DOC (in kg C year^−1^) as:

3 $$DOC=NPP*{FL}_{DOC}$$

4 $$POC=NPP*{FL}_{POC}$$

where ${FL}_{DOC}$ and ${FL}_{POC}$ are as above. We obtained estimates of the carbon content of seaweed from Duarte^95^, and the fractions of DOC and POC lost from Krause-Jensen and Duarte^34^.

#### Sequestration and Product Replacement

We estimated the potential for carbon sequestration (in kg CO_2_ year^−1^) as the sum of the sequestration and emission avoidance pathways:

5 $${C}_{Total} = {{C}_{Seq\cdot P}+{C}_{Seq\cdot A}+{C}_{Avoid}}$$

Total carbon sequestration (${C}_{Total}$) is the sum of passively sequestered carbon (${C}_{Seq\cdot P}$) via POC and DOC, actively sequestered carbon (${C}_{Seq\cdot A}$) via purposefully leaving or releasing harvested kelp into the marine environment, and carbon emissions avoided by replacing other products ${(C}_{Avoid})$. Carbon sequestration values are expressed as kg CO_2_ year^−1^. Calculation of the sequestration related values include a correction to account for the biological (e.g., respiration) and oceanographic (e.g., upwelling) processes which replace CO_2_ in surface waters and means the carbon sequestered in seaweed tissue does not have a one to one relationship to atmospheric drawdown of CO_2_^52,59^. Details on each of the pathways in Eq. 5 and the associated parameters can be found in the “Supplementary Methods”.

#### Emissions

We estimated total carbon emissions (in kg CO_2_e year^−1^) from the production and use of kelp as:

6 $${E}_{Total} = {{E}_{Nurs}+{E}_{Cap}+{E}_{Mat\cdot Trans}+{E}_{Maint}+{E}_{Seq}+{E}_{SW.trans}+{E}_{Proc}}$$

Total carbon emitted (${E}_{Total}$) is the sum of emissions from nursery operations (${E}_{Nurs}$), the production of capital equipment (${E}_{Cap}$), material transport (${E}_{Mat\cdot Trans}$), farm maintenance (${E}_{Maint}$), active sinking of kelp (${E}_{Seq}$), the transport of harvested kelp to port (${E}_{SW\cdot trans}$), and the processing of kelp into final products (${E}_{Proc}$). All emissions are expressed as kg CO_2_e year^−1^. This sub-model captures many of the primary sources of emissions, however other potentially important emissions such as those from waste management, product storage, or product transport could not be included due to insufficient information. We provide the details on the representation of the pathways underlying the emissions in Eq. 6 and the associated parameters in the “Supplementary Methods”.

### Parameter values

We obtained parameter values from the literature and through discussions with seaweed producers in BC and Alaska. In this region, the seaweed cultivars are dominated by fast-growing brown kelps (order Laminariales, including *Saccharina latissima*, *Alaria marginata*, *Nereocystis luetkeana, and Macrocystis* spp.). Kelp aquaculture in this region typically involves culturing kelp ‘seed’ (gametophytes and juvenile sporophytes) in a controlled nursery, which is then applied to floating longlines for a single cultivation and harvest period each year. Harvested kelp biomass is transported to primary processing (e.g., freezing, drying) facilities by boat, after which the biomass may travel onwards for additional processing.

Wherever possible, we gave preference to parameter values from local seaweed producers or literature values derived in the eastern North Pacific. For each parameter we defined a quantitative distribution when data were sufficient to provide a standard deviation or a minimum and maximum value. When data were insufficient, uncertainty was assessed qualitatively to define a distribution (e.g., ± 50%). We used normal (often truncated at zero), uniform, or triangular parameter distributions depending on the available information. Further discussion of parameter values and how they were derived is provided in the “Supplementary Material”.

For the model parameters that vary spatially (e.g., transport emissions between port and the farm site) we used a zonal approach. We calculated these parameters using area-weighted averages to account for the envisioned extent of kelp farms under each scenario (see building kelp farming scenarios below and “Supplementary Material”).

### Engagement with regional kelp producers

We engaged with kelp producers from California to Alaska to parameterise the model and develop appropriate scenarios. We conducted unstructured interviews and distributed a questionnaire (see Supplementary Table S6), with a focus on kelp production rates, and product and emission pathways. We received five responses with varying levels of detail. Some respondents declined to answer specific questions for proprietary reasons, while several provided detailed responses regarding production and maintenance. Less information was provided on emissions as data were often not available or hard to access. One individual declined to participate because the level of detail requested was too specific.

### Building kelp farming scenarios

We used a combination of survey responses and unstructured interviews with kelp producers in BC and Alaska to ground our scenarios in species profiles, farm footprints, production rates, emission sources, and seaweed fates common to the temperate eastern North Pacific. Our first two scenarios characterise the state of affairs reported by current producers in BC, expanded to 507 km^2^ of suitable area in shallow waters, and assuming farms remain close to coastal communities. The first scenario (*Local-No Harvest*) assumes all kelp is left in the water, akin to natural kelp beds. We included this scenario as a point of comparison for the remaining scenarios. The second scenario (*Local-Products)* is identical to the first in terms of spatial extent and production, but assumes kelp is harvested and used for various purposes as reported by local producers (Table 1).

Our third (*Expanded*) and fourth (*Expanded-Optimized*) scenarios represent expansion of the industry to 1210 km^2^ of shallow and deep waters in close proximity to communities. Kelp fates for both scenarios resemble those used in the *Local* scenarios but are further diversified to represent emerging new markets for seaweed biomass (e.g., perhaps driven by the saturation of existing markets; Table 1). The *Expanded* scenario uses the kelp production rates reported by producers (as with the first two scenarios), while the *Expanded-Optimized* scenario uses production rates from the literature which are substantially higher than those currently reported by producers in BC and Alaska. The literature values include production rates from field studies and modelling in various temperate locations (see Table S5, “Supplementary Material”), and reflect the potential for optimization as the industry develops.

The fifth and final scenario (*Techno Industrial*), represents a maximal approach, assuming kelp farming covers 5681 km^2^ extending to deep and shallow areas across the coast, regardless of proximity to communities. In this scenario, half of all kelp produced is transported and sunk in deep water with the remainder for replacement products (Table 1). As with the *Expanded-Optimized* scenario, production rates are assumed to be optimized, and are based on values reported in the literature.

### Spatial extent of kelp aquaculture

To estimate the area available for kelp aquaculture in BC under each scenario we used suitability restrictions based on depth, substrate, proximity to communities, and existing human uses. All spatial calculations were performed using ArcGIS 10.3^96^.

Current kelp farms in BC and Alaska have a small footprint (up to 8 hectares or 20 acres each) and are largely focused on value-added production. They are generally limited to shallower depths and soft or mixed substrates to facilitate anchoring, and also tend to be located close to coastal communities to facilitate logistics (*Local-No Harvest* and *Local-Products* scenarios). We assumed that with additional investment, farms could operate in deeper waters (though with higher emissions), greatly expanding the potential footprint on the coast both in close proximity to communities (*Expanded* and *Expanded-Optimized* scenarios) and in more remote areas (*Techno Industrial* scenario).

We identified suitable depths using a 100 m bathymetry^97^. We excluded areas less than 15 m to ensure sufficient farm depth, and greater than 200 m (the approximate depth of the shelf break). Shallow waters were defined as between 15 and 75 m depth, while deeper waters were 75–200 m. We identified suitable substrate based on Gregr et al.^98^ and defined all soft-bottom areas as suitable. To represent proximity to existing coastal communities, we buffered populated locations^99^ to 25 km to define marine zones in close proximity to communities. Depth, substrate, and proximity restrictions were applied based on feedback from local producers.

We estimated the size of incompatible human use areas (i.e., those dominated by transportation, commercial fishing, recreation, and protected areas) based on footprints of cumulative impacts in the coastal environment^100^. We defined areas incompatible with kelp farming as those with existing human uses above a minimum cumulative effect score. For the coastal footprint, we selected a minimum threshold (2.5) as this provided some exclusion around larger coastal communities in BC.

In addition to these spatial restrictions, we further limited kelp aquaculture to 10% of the total available area to account for additional spatial restrictions not reflected in our calculations (e.g., wave exposure, nutrient limitation, additional competing uses) and the significant logistical challenges faced by many areas of BC’s remote coast. Other modelling efforts have made similar but less conservative assumptions (e.g., Spillias et al.^17^ assume 50% suitability). Our approach is intentionally conservative, reflecting the important environmental conditions and local contexts not accounted for in our calculations.

### Sensitivity analysis

We used a sensitivity analysis to assess the relative contribution of different model parameters to the uncertainty in our model estimates. We assessed model sensitivity at the resolution of each sub-model (Production, Sequestration and Products, Emissions) and for the individual parameters within each sub-model. We assessed sensitivity using Monte Carlo simulations where the parameter(s) of interest were sampled with uncertainty while holding all other parameters at their central estimate. We used 10,000 runs to describe sub-model sensitivities and 1000 runs for individual parameter sensitivities.

We were primarily interested in the effect of parameter uncertainty on the predicted net climate benefit (Tg CO_2_e year^−1^). We converted this to a relative value by dividing the estimate from each sensitivity analysis by the predicted net climate benefit with no uncertainty (i.e., with all parameters held at their central estimate). This allowed comparisons between sensitivity analyses as well as to the full model, providing insight into the uncertainty in each sub-model as well as in specific parameters. Sensitivity analysis results are scenario-specific, varying based on the parameters used and the selected kelp fates.

## Economic assessment

We conducted a simple economic assessment using costs for seaweed production, transport, processing, and the value of seaweed products from previously published techno-economic models of seaweed aquaculture^38,52^ to provide insight into the economic feasibility of our five scenarios. This assessment considered overall cost for seaweed production, cost of transporting seaweed to port or sinking locations, and cost of processing seaweed into a product (see “Supplementary Material” for individual parameter values). Product values were calculated for each of the three product types and offset against costs to calculate net costs. To specifically examine the cost of climate benefits from each scenario we report net cost per TgCO_2_e^−1^ sequestered or avoided, as well as total cost, total product value, and net cost, all in Canadian dollars. We did not consider the potential value of carbon credits for sequestration or avoided emissions, as it is not clear which (if any) of the pathways explored here might be suitable for carbon credits.

Local information on costs and product values for BC were not available, necessitating the use of estimates from other regions or global assessments. Similarly, the cost for seaweed production includes both fixed costs (equipment, license fees, financing, etc.) and operating expenses (nursery costs, vessel contracting, labour, consumables etc.)^38^, but disaggregating these costs and calculating them for the BC context was not possible with the information available. Thus, while this assessment provides insight into the relative economic feasibility of our different scenarios and how they may compare to other climate mitigation strategies, it may differ from local socio-economic contexts.

### Supplementary Information

Supplementary Information.

## Data Availability

The data and equations needed to reproduce this analysis are contained in the supplementary materials and also available on GitHub (https://github.com/CamBullen/Bullen-et-al.-2024-SciRep).
